# Contingent Valuation: A Pilot Study for Eliciting Willingness to Pay for a Reduction in Mortality From Vaccine-Preventable Illnesses for Children and Adults in Bangladesh

**DOI:** 10.1016/j.vhri.2020.10.004

**Published:** 2021-05

**Authors:** Deborah Odihi, Gatien De Broucker, Zahid Hasan, Sayem Ahmed, Dagna Constenla, Jasim Uddin, Bryan Patenaude

**Affiliations:** 1 International Vaccine Access Center, Johns Hopkins Bloomberg School of Public Health, Department of Internaional Health, Baltimore, MD, USA; 2 International Centre for Diarrheal Disease Research, Bangladesh, Dhaka, Bangladesh; 3 GlaxoSmithKline Panama City, Panama

**Keywords:** Bangladesh, contingent valuation, dengue, economic burden, stated preference methods, vaccine-preventable diseases, value of statistical life (VSL)

## Abstract

**Objectives:**

The contingent valuation (CV) method elicits willingness to pay (WTP) for calculating the value of statistical life (VSL). CV approaches for assessing VSL are uncommon in many low and middle-income countries (LMICs). Between 2008 and 2018 only 44 articles utilized WTP in a health-related field and of these only 5 (11%) utilized CV to assess the WTP for a mortality risk reduction. We elicit WTP estimates and compute VSL using the CV method in Bangladesh.

**Methods:**

The pilot study was primarily aimed at developing best practice guidelines for CV studies in LMICs to get more robust WTP estimates. To this end, we explored three methodological a) Varying the name of the intervention, keeping all other characteristics constant; b) varying the effectiveness of the health intervention and c) offering an overnight period to think about the WTP scenario. The survey was administered 413 randomly selected participants. VSL was for a 1/3000 mortality risk reduction.

**Results:**

We had more males (54%) than females (46%) and the mean annual self-reported income was $5,683.36. Mean VSL is $11,339.70 with a median of $10,413. The ratio of child: adult WTP is approximately 1 by both gender and age category. The vaccine intervention had the largest amount of $0 WTP and protest responses (52% and 58% respectively). 93% of the participants were able to describe (teach-back) the vaccine effectiveness using their own family as an example.

**Conclusion:**

Our study provides empirical evidence on how to better generate CV surveys to produce more robust WTP estimates.

## Introduction

Averted medical costs, out-of-pocket expenditures, and productivity loss, together called the cost of illness, are used ubiquitously to estimate the value of vaccines and immunization strategies in different contexts.^1^, ^2^, ^3^, ^4^, ^5^, ^6^, ^7^, ^8^ Using cost of illness as a measure of the economic impact of vaccination likely undervalues the broader benefits of vaccination, because it excludes nonpecuniary gains in welfare for the individual and the involved community.^9^^,^^10^ Alternatively, the value of a statistical case or value of statistical life (VSL), derived from studies of individuals’ willingness to pay (WTP) for small changes in their own morbidity risk or mortality risk, respectively, can be used to estimate the value of vaccines. These approaches capture an individual’s preferences for health improvements and may encompass perceived benefits, impact on earnings, and improved happiness or quality of life.^11^^,^^12^ VSL measures are useful for economists and policy makers using benefit-cost analysis to prioritize different life-saving interventions.^13^ The WTP value for computing VSL can be elicited through contingent valuation (CV) studies or discrete choice experiments. Although common in many developed settings, CV approaches for WTP and studies eliciting empirical VSL are uncommon in many low- and middle-income countries (LMICs) because of lower numeracy and literacy rates.^14^ In fact, between 2008 and 2018 only 44 articles from Gavi-eligible Asian countries used WTP in a health-related field, and of these only 5 (11%) used CV to assess the WTP for a mortality risk reduction. In this study, we pilot several CV survey methodologies to elicit WTP estimates and compute VSL in Bangladesh. Our primary goal is to provide evidence on the impact of various survey and methodological choices for CV on elicited WTP and VSL and to expand the literature on stated preference elicitation in LMICs.

## Materials and Methods

### Development of the Survey

This study was performed in collaboration with the International Center for Diarrheal Disease Research, Bangladesh (icddr,b). The survey was designed using a 5-stage iterative process decided on by an expert panel to mitigate challenges and gaps identified during the presurvey literature review. The stages included: (1) a literature review of CV surveys in LMICs; (2) interviews with health economists conducting WTP surveys; (3) a focus group with Bangladeshi students at Johns Hopkins University on the appropriateness of the questions; (4) consultation with experts on technical aspects of the survey; and (5) a focus group hosted by icddr,b, Mohakhali, Dhaka to finalize the survey and ensure setting appropriateness, participant understanding, and accuracy of translation. Table 1 highlights changes made to the survey at each stage.

**Table 1:** Survey changes after the iterative process and focus group discussions.

Initial survey component	Final survey component	Reason for change
**Respondent characteristics question:** Asked all participants of they had children	Unmarried women were not asked if they had children.	It is not culturally appropriate to ask unmarried women whether they have children. This question was updated after the focus group discussion with Bangladeshis in the Baltimore area.
**Portion of the vignette and visual aid for teaching risks and probabilities:** Scenario 1 The square grid below (grid 1) contains 100 small squares within the larger square. One percentage point is a 1 in 100 possibility of something happening. Let us explain this concept using some examples. Imagine that your neighbor Ali, who lives with his wife and 2 children, is a farmer that plants wheat on his farm. There was a flood and the wheat crops on Ali’s farms are at risk of being destroyed by the flood. The grid below represents Ali’s farm and currently shows that the only crop Ali currently plants is wheat. So let us say that he has 100 wheat crops as represented by the 100 small squares with wheat plants in the grid. The probability that Ali will experience any loss related to jute plants is 0, simply because Ali does not grow any jute on his farm. This means that there is a 0 in 100 chance or 0% chance that Ali will lose any jute plants because he has none	In addition to the symptoms caused by dengue, dengue can also lead to death. We will be looking at some diagrams below to explain the concept of percentages and death risks. *Scenario 1* Imagine that there are 20 people in your community, and you are one of those 20 people. There is an outbreak of dengue in your community, and all 20 of you are at risk of getting dengue. The grid below shows 1 person colored in red, and everyone else colored in yellow. This person colored in red dies from the dengue outbreak, and it can be anyone in the community, including yourself. Since there are 20 people in your community, and 1 person dies, this shows a 1 in 20 risk of death. This 1 out of 20 risk is the same as 5%.	The entire initial vignette was changed after feedback from experts and the focus group with Bangladeshis in the Baltimore area. The feedback explained that the vignette was confusing and difficult to follow. Using some of the suggestions and feedback from the focus group and expert opinion, we updated the vignette.
**Vaccine Effectiveness diagram**		The diagram was simplified based on expert input to focus on the vaccinated individuals only. This helped improve the conciseness and understanding of the accompanying vignette.
**Framing of the unnamed health intervention WTP section:** It was called an unnamed preventative health intervention throughout the survey	After the focus group discussion in Bangladesh, we added a hint to the survey for the data collectors to call the intervention a “preventive medical procedure or preventive public health intervention” if the respondent insisted on being told the name of the intervention.	Our colleagues and local stakeholders expressed during the focus group in Bangladesh that people will likely insist on being told what exactly the intervention is, so the definition given would need to be standardized across all data collectors.
**Income and socioeconomic status information:** The question on health spending asked for health spending in the previous month	The question was changed to ask for health expenditure in the last 3 months	This change was made after the focus group discussion in Bangladesh. We had discussions on how the respondent may not have had a reason to seek health in the past month, so a 3-month period would be more feasible
**Income and socioeconomic status information:** The question on household food expenditure was asked as a single aggregate question	We disaggregated household spending into line items such as housing and utilities, education, clothing, and other spending.	During the focus group discussion in Bangladesh, we decided that it was better and easier for respondents if the expenditure question was disaggregated into different components.
**WTP section** The WTP section ended if individuals opted to have a free vaccine instead of paying for it.	We included a question that probed why the respondents wanted a free vaccine instead of having to pay for one.	The expert review offered this suggestion as a means to better understand and check if income was the only reason why people would not want to pay for a vaccine, and thus probe for protest responses.
**WTP section** This section did not initially have a question that asked respondents how they would accommodate the payment for the vaccine.	We included a question that asked the respondent what adjustments would be made to their budget if they had to pay for the vaccine, as they thought through the WTP section of the survey.	To make the scenario as realistic as possible, we added a question on budgetary adjustments necessary to accommodate the payment for the vaccine. This change was effected after the expert review and was in line with evidence found in the literature.^15^
**WTP section** The start bid, effectiveness for the intervention, and time-to-think method was varied for different surveys.	We included a randomizer in the coding of the surveys so that start bid, intervention effectiveness, and time-to-think versions of the survey would be random, instead of being decided on by the data collectors.	During the focus group discussion, the data collectors expressed concern about deciding the various versions of the survey and asked that it be randomized to avoid error and bias when deciding the version of the survey to use.
**WTP section** When explaining the vignette disease, dengue, before eliciting WTP value from respondents, we took time to discuss the symptoms of dengue and the transmission of dengue.	We made sure to include death as a symptom of dengue and also were more intentional with including death in the vignette.	After the expert review, we decided that we should have more statements about the risk and probability of death so that the respondents would be even more aware that their WTP value was for a mortality risk reduction as opposed to a morbidity risk reduction. This was necessary because we intended to calculate VSL.
**Throughout the survey** We had questions throughout the survey that had the option “I don’t know” in case the respondent could not answer a question.	We removed the option “I don’t know” and instead increased the number of choices that the respondent could choose from, and we added then option of “other” whenever necessary.	This change was made after the expert review as a means to get respondents to think about the questions carefully and offer thoughtful responses.

VSL indicates value of statistical life; WTP, willingness to pay.

After our literature review and expert panel discussions, we decided to examine 3 specific methodologies. The choices were hypothesized to influence a respondent’s WTP and they include: (1) varying the name of the interventions with exact same attributes (ie, a vaccine, a pill, and an unnamed health intervention); (2) varying the health intervention effectiveness (50% or 90% effective); and (3) an extended overnight period to think about the WTP for the intervention. These choices were randomly varied across the surveys, and their impacts on WTP are analyzed and discussed in the results section.

The intervention name was varied to examine whether anchoring bias could be avoided by having different names for the same intervention, as vaccines are free in Bangladesh, and also to examine how much individuals consider information other than the presented information, such as market prices of related products, in their decision making.^17^, ^18^, ^19^, ^20^, ^15^ Dengue was the hypothetical disease in the vignette because it carries a positive mortality risk, is well understood in the setting, is vaccine preventable, and does not have an available vaccine in Bangladesh, which was important to minimize anchoring risk.

The varied intervention effectiveness served as a validity test to confirm whether people understood the attributes of the health intervention, as one of the most documented challenges with performing CV studies is ensuring that respondents understand the quantitative portion of the survey.^15^^,^^21^, ^22^, ^23^ Varying effectiveness while holding other attributes constant allows us to test whether individuals understand what they are paying for.

The time-to-think (TTT) method was implemented because literature on the TTT method demonstrates that incorporating it tends to give more conservative and potentially realistic WTP estimates as participants have more time to think about their choices and can discuss with other household members.^24^, ^25^, ^26^, ^27^, ^28^ Table 2 summarizes all the methodological choices and the rationale for these choices.

**Table 2:** Methodological choices for the contingent valuation (CV) pilot study and the rationale behind these choices.

Methodological choice	Details	Rationale
Name of the intervention	The health intervention was called 1 of 3 names: a vaccine, a pill, or a health intervention (unnamed). All interventions had identical attributes.	The name of the intervention was varied to confirm whether changing the name of the intervention could prevent anchoring bias. Anchoring bias occurs when a respondent bases their WTP value similar to the price of another similar good. In the Bangladeshi scenario where many vaccines are free of charge, people might anchor their WTP estimates on 0 Bangladeshi taka.^6^^,^^10^ This methodological choice was also explored to check and capture people’s preferences for how they would like the intervention to be delivered. We wanted to see if people preferred a different method of delivery from the traditional methods of delivery.
The effectiveness of the intervention	The effectiveness of intervention was either 50% effectiveness or 90% effectiveness.	This was done as a validity test to check whether people truly understand the attributes of the health intervention presented to them. The WTP for an intervention with a higher effectiveness should be higher.
Overnight, time-to-think (TTT)	Some participants will be given an overnight period to think about the information provided to them before responding to the WTP portion of the study.	Looking through the literature, some studies have suggested that people have lower WTP estimates with an extended time to think because they are more able to consider the budgetary implications of paying for the vaccine, and they also get a chance to discuss with other household members before making a decision.^1^, ^2^, ^3^, ^4^ This could allow for more robust and realistic WTP estimates.

WTP indicates willingness to pay.

### The Survey Instrument

The final survey instrument had 4 sections: (1) respondent identification and characteristics, (2) household demographics, (3) socioeconomic status and income, and (4) contingent valuation and WTP. The demographic information and respondent characteristic questions were asked ahead of the WTP question to devote more time to explaining the CV scenario. WTP was elicited using the bidding-game format followed by an open-ended question. The literature suggests that combining these 2 methods reduces starting point bias and incentive incompatibility, and provides a more accurate range of WTP estimates.^29^, ^30^, ^31^, ^32^, ^33^ The start prices were randomized and increased or decreased by 20% depending on the participant’s first response. The WTP question was as follows: “Are you willing to pay X Bangladeshi taka for a dengue vaccine that reduces the risk of death by 1/3000?” Data collectors each rotated between the different names for the health intervention (vaccine, medication, and unnamed health intervention), and the price, vaccine effectiveness, and time-to-think versions were randomly generated for the data collectors using the KoBo toolbox software.

To aid understanding the CV scenario, we used vignettes (short stories that explain a concept) with visual aids and the cognitive interview teach-back method. The vignettes and images illustrated the vaccine-preventable disease, explained the numerical risk and probabilities, and elicited WTP values for children in the non–caretaker population. The vaccination vignette was adopted from the literature; however, it focused on vaccinated individuals, not on individuals vaccinated in relationship to the rest of the community, because Bangladeshis are familiar with getting vaccinated.^28^^,^^34^, ^35^, ^36^, ^37^ This local familiarity kept the vignette short and easily understandable, as recommended by experts involved in the survey development.

The cognitive interview “teach-back” methodology requires patients to re-explain presented information in their own words.^38^^,^^39^ It provides a mechanism for knowing how much of the information presented is absorbed and understood. We asked respondents to describe the vignette scenario using their own household as an example. The data collectors then evaluated the accuracy of responses based on 2 criteria: (1) the respondent indicated that 50% or 90% of household members were protected, and (2) unprotected household members are just as likely to be infected with dengue as other community members without exposure to the health intervention. If the first response was incorrect, the data collector explained the information only once more and then evaluated the respondent’s understanding and proceeded with the study irrespective of understanding. Other surveys in the literature evaluated participants’ understanding using quizzes.^24^^,^^27^^,^^28^ However, clinical evidence shows that people recall and retain information better with the teach-back method; thus we adopted this approach.^40^, ^41^, ^42^, ^43^, ^44^

Quarterly and yearly income was collected and converted for participants with seasonal income. On average, the interviews took 1 hour and 15 minutes per participant to complete for the non-TTT surveys, and the TTT surveys took about 2 days to complete.

### Study Population and Sample Size

The target population was the general population at the Dhaka Hospital, icddr,b, and the Dhaka Shishu (Children) Hospital. This included patients and nonpatient caretakers with or without experience of the vignette disease, dengue. A caretaker in this pilot study was defined as someone who has lived in the same household as the child for whom they are responsible for at least 12 months. If the child is younger than 12 months, the caretaker must have lived with the child for the child’s entire life. The participants of the study were patients aged 18 years or older who were willing to participate. The sample size was a convenience sample of 413 participants quasi-randomized to every 10th patient at both hospitals.

### Data Collection Procedure

Written informed consent was obtained from all enrolled participants, and the study was approved by the Johns Hopkins University (ID: IRB-7256) and icddr,b (ID: PR-16067) institution review boards. Data were collected using the KoBo toolbox database software.

### Data Analysis

We assessed VSL using 2 approaches. In the first, we calculate VSL using the WTP for each risk reduction, and in the second, we calculate a backed-out VSL by comparing responses to 2 WTP questions. The only difference between the 2 questions for the backed out VSL is that the first states that the hypothetical health intervention reduces mortality risk by 1 in 3000, and the second states that the hypothetical health intervention reduces the risk of death by 1 in 1000. Because the effectiveness of the vaccine against dengue contraction and all other methodological features are the same in both scenarios, the only difference is a mortality risk change, and the attribution to dengue cancels out. We can therefore take the difference in the monetary WTP values elicited and divide by the risk change to compute a backed-out measure of VSL.

$$VSL=\frac{1\;}{\text{Risk reduction }}\;\times \;WTP$$

$$Backed\;out\;VSL=\frac{\left(\text{WTP}\frac{1}{1000}\text{risk reduction}\right)-\left(\text{WTP}\frac{1}{3000}\text{risk reduction}\right)\;}{\frac{1}{1000}\;-\;\frac{1}{3000}}$$

Owing to skewness of the data, Wilcoxon nonparametric tests for comparison of median values of WTP and VSL were used instead of standard *t* tests. Additionally, linear regression analysis on both the natural units and log scale were conducted to compare differences in elicited VSL between test methodologies. All monetary values were elicited in Bangladeshi taka and converted to 2019 US dollars at a rate of 1 Bangladesh taka = 0.01157 US dollars.^45^ All statistical analyses were performed in Stata, version 15.1.

Furthermore, the 2 different risk reduction questions were also employed as scope tests to examine the sensitivity of individual WTP to changes in risk and to ensure that it is consistent with economic theory and an appropriate understanding of the questions (higher risk reduction correlated with higher WTP). Protest responses were identified if participants reported both a $0 WTP and responded that vaccines or other interventions being available for free in the health system was the rationale for their response.

## Results

### Demographics

In our sample, 54% of participants were male, and 46% were female; 89% of our sample participants were married, whereas 9% were single and 1% divorced. The age range for the participants was 18 to 70 years. The average self-reported household monthly income was $473.53 (standard deviation [SD] $506.01), which corresponds to an annual income of $5683.36 (SD $6072.12). Per capita gross domestic product (GDP) of Bangladesh is $1698.26, so mean income was approximately 3.3 times higher than per capita GDP.^46^

#### WTP and VSL

The average WTP for a 1/3000 and 1/1000 risk reduction for men was $4.24 and $5.63, respectively, and $3.23 and $4.94, respectively, for women. The average VSL for a 1/3000 and 1/1000 risk reduction was $12 706.14 and $9676.55 for men and $5633.99 and $4937.91, respectively, for women, as shown in Table 3. Men had a statistically significantly higher WTP and income than women (Wilcoxon test *P* value: 0.000 and 0.000, respectively). Men also had a higher mean WTP for children than women, at $5.78 and $4.41, which corresponds to VSLs of $5776.55 and $4937.91 for men and women, respectively. This difference was statically significant (Wilcoxon test *P* value: 0.0004). The ratio of child-to-adult WTP is approximately 1 by both gender and age category. This finding implies that the WTP child-to-adult ratio in Bangladesh might be different from the prevailing 1.5:1 ratio found in high-income country settings.^47^

**Table 3:** Mean WTP and VSL by sex.

Sex	Mean monthly income	Mean WTP (1/3000 risk reduction)	Mean WTP (1/1000 risk reduction)	Mean VSL value (1/3000 risk reduction)	Mean VSL value (1/1000 risk reduction)	Mean VSL value for children (1/1000 risk reduction)	WTP ratio (child: adult)
Male	$552.71	$4.24	$5.63	$12 706.14	$5633.99	$5776.55	1.11
Female	$378.51	$3.23	$4.94	$9676.55	$4937.91	$4411.86	1.17

VSL indicates value of statistical life; WTP, willingness to pay.

Approximately 25 individuals (6%) of the overall sample had a WTP of $0, and 12 (48%) of the $0 WTP responses were deemed to be protest responses. Protest responses were identified if participants refused to pay because vaccines or other interventions in the health system were free, instead of reporting an inability to pay. There was no difference in mean or median WTP with or without the protest responses included (Wilcoxon test *P* value: .559).

### TTT Surveys

Follow-up for the TTT survey was conducted through telephone calls. If respondents declined the TTT version, we asked whether they would prefer to complete the survey in the current sitting. The TTT survey was received by 46% of the participants; 33% declined, and data collectors proceeded to complete the survey in the same day. Men and women were similarly likely to refuse TTT (33 men vs 29 women). Consequently, we had 30% TTT surveys and 70% regular surveys. The average WTP for 1/3000 and 1/1000 risk reductions for the non-TTT surveys was higher than that of the TTT surveys at $3.81 and $3.28 for TTT surveys and $5.62 and $4.32 for non-TTT surveys, respectively. This more conservative result from the TTT survey is expected based on evidence from the literature; however, none of these differences were statistically significantly different from non-TTT values (Wilcoxon test *P* value between the TTT and regular surveys was .169 and .105 for the 1/3000 and 1/1000 risk reductions, respectively).^24^, ^25^, ^26^, ^27^, ^28^

### Varying the Name of the Intervention

The intervention name was varied between a vaccine, a medication, or an unnamed health intervention to evaluate its effect on WTP. For the unnamed health intervention, the participants were asked to imagine a health intervention of their choice and then give a WTP value for that intervention. There were 135 vaccine surveys, 136 medication surveys, and 142 unnamed health intervention surveys. The WTP and VSL for the unnamed intervention was lowest, followed by the vaccine intervention, and the medication intervention yielded the highest VSL values. The vaccine intervention also had the highest amount (52%) of $0 WTP values. The mean VSL values for the 1/3000 risk reduction were $11 081.24, $12 151.37, and $10 762.73 for the vaccine, medication, and unnamed health intervention, respectively, as shown in Table 4.

**Table 4:** VSL (1/3000 risk reduction) for the different names of the health intervention.

Intervention type	Mean VSL	Mean VSL (international dollars)	Median VSL	Median VSL (international dollars)	Number of $0 WTP responses
Vaccine	$11 081.24	$31 218.81	$10 413	$29 336.20	12
Medication	$12 151.37	$34 233.65	$10 413	$29 336.20	3
Intervention	$10 762.73	$30 321.48	$8677.5	$24 446.83	8

VSL indicates value of statistical life; WTP, willingness to pay.

### Regression Models

**Figure 1:**
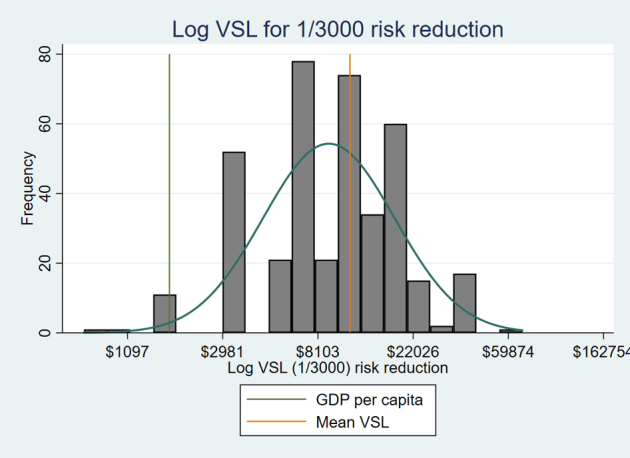
Log VSL (1/3000 risk reduction) graph showing the mean VSL and GDP per capita in Bangladesh.

We ran multiple linear regressions with WTP and VSL as our dependent variables on the log and dollar scale. We controlled for monthly income, age, gender, intervention effectiveness, overnight time to think, and the name of the intervention in the survey. These independent variables were chosen to verify the theoretically predicted positive relationship between income and WTP and also to examine the relationship between age, sex, and VSL to document how these demographic factors relate to VSL. Furthermore, we ran regressions with our methodological choices to confirm how these variables influenced the WTP and VSL values, controlling for the other demographic factors. Figure 1 shows the distribution of the log VSL for a 1/3000 risk reduction, highlighting the mean VSL and current GDP per capita in Bangladesh.

GDP indicates grosss domestic product; VSL, value of statistical life.

In all models, income had a statistically significant positive relationship with WTP and VSL. On average, a $1 increase in monthly income increases WTP for a 1/3000 risk reduction by $0.00075 (*P* = .004), holding all else constant as seen in Table 5. On the natural log scale, a 10% increase in monthly income is associated with a 2.25% (*P* = .000) increase in WTP for a 1/3000 risk reduction, holding all else constant. Age did not have a significant relationship with either VSL or WTP whether it was included as continuous or categorical variable. The TTT version did not have a statistically significant relationship with VSL and WTP, most likely because of the limited sample. The intervention effectiveness also did not have a significant relationship with WTP and VSL in all models. The medication survey had a positive relationship with VSL and WTP in all models, explaining that, on average, people are willing to pay more for medications, all else constant. Comparing the medication survey to the unnamed intervention, people were willing to pay $1.36 (*P* = .060) more for the medication survey for a 1/1000 risk reduction.

**Table 5:** The estimated WTP and VSL (value of statistical life) for a dengue intervention that reduces the risk of mortality from dengue by 1/3000 or 1/1000 on the dollar and natural log scale.

Variables	WTP 1/3000 risk		WTP 1/1000 risk		VSL 1/3000 risk		VSL 1/1000 risk	
	Coefficients on the dollar and natural log scale							
	$	Ln	$	Ln	$	Ln	$	Ln
Monthly Income†	0.000750∗∗∗	0.225∗∗∗	0.00107∗	0.203∗∗∗	2.250∗∗∗	0.225∗∗∗	1.071∗	0.203∗∗∗
	(0.000262)	(0.0470)	(0.000587)	(0.0506)	(0.785)	(0.0470)	(0.587)	(0.0506)
Gender‡	–0.878∗∗∗	-0.182∗∗	–0.527	–0.172∗∗	–2634.0∗∗∗	–0.182∗∗	–526.9	–0.172∗∗
	(0.276)	(0.0716)	(0.617)	(0.0769)	(828.0)	(0.0716)	(617.2)	(0.0769)
Age >27§	–0.176	0.00634	–0.409	–0.0537	–526.9	0.00634	–409.0	–0.0537
	(0.278)	(0.0712)	(0.621)	(0.0765)	(833.3)	(0.0712)	(621.0)	(0.0765)
Intervention Effectiveness‖	0.0163	0.000360	–0.498	–0.0229	48.79	0.000360	–497.6	–0.0229
	(0.266)	(0.0682)	(0.595)	(0.0733)	(798.3)	(0.0682)	(595.1)	(0.0733)
Time to think¶	–0.266	0.0133	–0.683	0.00627	–798.7	0.0133	–683.4	0.00627
	(0.295)	(0.0757)	(0.660)	(0.0812)	(885.8)	(0.0757)	(660.0)	(0.0812)
Medication survey#	0.424	0.00476	1.357∗	0.0506	1271.2	0.00476	1357.2∗	0.0506
	(0.321)	(0.0825)	(0.719)	(0.0886)	(964.2)	(0.0825)	(719.4)	(0.0886)
Vaccine survey††	0.0638	–0.00683	0.492	0.0467	191.4	–0.00683	491.6	0.0467
	(0.328)	(0.0842)	(0.733)	(0.0903)	(983.7)	(0.0842)	(732.5)	(0.0903)
Constant	3.823∗∗∗	–0.141	5.117∗∗∗	0.287	11470.1∗∗∗	7.865∗∗∗	5117.4∗∗∗	7.195∗∗∗
	(0.395)	(0.299)	(0.885)	(0.322)	(1184.5)	(0.299)	(884.9)	(0.322)

*Note.* ∗∗∗, ∗∗, and ∗ denote statistical significance at the 1%, 5%, and 10% levels, respectively. Standard errors are in parentheses.

Ln indicates the natural log; WTP, willingness to pay.

† The income variable is a continuous variable for monthly income.

‡ The gender variable is a dichotomous variable that is coded 0 for males and 1 for females.

§ The age variable is a categorical variable that is coded 0 for participants 27 years and younger, and 1 for participants older than 27 years.

‖ The intervention effectiveness variable is a dichotomous variable that is coded 0 for 50% effectiveness and 1 for 90% effectiveness.

¶ The TTT variable is a dichotomous variable coded 1 for TTT surveys and 0 for non-TTT surveys

# The medication survey variable is a dichotomous variable coded 1 for surveys with medication as the intervention and 0 for surveys with vaccines and unnamed health interventions.

†† The vaccine survey variable is a dichotomous variable coded 1 for surveys with vaccine as the intervention and 0 for surveys with medication and unnamed health interventions.

### Scope Tests

Scope tests are used to check whether respondents understood the different risks by confirming that people value different larger risk reductions more, in accordance with economic theory. Overall, scope held for a majority of the participants (62%). Interestingly, 38% of the survey participants were not willing to pay more for the 1/1000 risk reduction when compared to the 1/3000 risk reduction. They valued both risk reductions the same. Excluding the protest responses, 35% of the respondents valued both risk reductions the same, and 65% valued the 1/1000 risk reduction larger than the 1/3000 risk reduction. No participant valued the 1/1000 risk reduction less than the 1/3000 risk reduction, showing that they understood the relative magnitude of the risks presented to them in the survey. Furthermore, 93% of the participants re-explained the vaccine effectiveness and risk concept correctly using their own family, showing that, overall, the respondents understood the risk and effectiveness concepts.

## Discussion

Our primary goal was to examine the effect of the methodological choices on empirical estimates of WTP and corresponding VSL in a LMIC setting. We were also interested in understanding what demographic and socioeconomic factors influence people’s WTP in Bangladesh. One of our main findings relates to the naming of our hypothetical intervention. The vaccine intervention had the largest amount of $0 WTP responses. Vaccines are free in Bangladesh in the public sector; thus, more participants appear to have anchored their WTP on $0 or had protest responses for the vaccine survey (52% and 58% of all $0 WTP values and protest responses, respectively, were found in the vaccine survey).^16^, ^17^, ^18^, ^19^, ^20^ Moving away from calling the intervention a vaccine by having an unnamed intervention in the survey was also challenging to implement in the field. Respondents could not understand why they should imagine or pay for a health intervention that had no specific name and requested that the intervention be given a name. To ensure data standardization, we called the unnamed intervention a “medical procedure” or “public health intervention,” upon request by participants. Data collectors explained that health professionals inform patients on necessary health services to maintain good health, so participants are not familiar with suggesting health interventions for themselves. This resulted in respondents having the lowest WTP for an unnamed health intervention, when compared to both vaccines and medication.

A second finding concerns the feasibility of a TTT approach in Bangladesh. We found the TTT method challenging to implement in Bangladesh, even in Dhaka.^24^, ^25^, ^26^, ^27^, ^28^ In fact, 33% of the randomized TTT surveys were completed in the same day because respondents did not have cell phones. In the future, it would be important to explore other feasible follow-up mechanisms. Respondents could also be given a few hours to think through the scenario prior to asking the WTP question while still at the health facility, to eliminate follow-up via cell phones.

Household incomes are statistically significantly associated with higher WTP and VSL. This is consistent with economic theory where a higher ability to pay should translate into higher willingness-to-pay, on average. We also find that, even after controlling for income, gender is significantly associated with WTP. Here we find that women have lower WTP and corresponding VSL than men. In some studies women have been found to exhibit greater social preferences and higher willingness-to-invest in health than men, so the finding that women are, on average, willing to pay less than men, even after controlling for income, is inconsistent with our initial expectations. We also found no relationship between age and WTP or VSL, regardless of whether it was included as a continuous or categorical variable in the model, after controlling for income, gender, effectiveness of the intervention, overnight time to think, and the name of the intervention in the survey. This adds evidence to the debate over whether or not age is systematically related to differences in WTP for health risk reductions. Furthermore, our findings show that some people valued a 50% effectiveness higher than a 90% effectiveness, with a negative coefficient on intervention effectiveness, as seen in Table 4. However, we suspect that this was the case because in the 90% effectiveness group, the largest proportion of the surveys was the unnamed intervention survey, and in the 50% group the largest proportion was the medication survey. As seen in Table 5, the unnamed intervention had the lowest WTP, and the medication had the highest WTP.

Regarding scope, 35% of our participants valued both risk reductions (1/1000 and 1/3000) the same, but no one valued 1/1000 less than 1/3000. It is possible that the difference in risk reduction between the 2 questions was too small to be valued differently by participants. This raises a question about what threshold for a difference in risk reduction is necessary to be viewed as a “different economic good” to individuals. The lack of sensitivity to risk changes may also indicate that risk changes may not be viewed in continuous units but instead as discrete risk intervals where WTP is constant until a sufficiently large jump occurs. This behavior is observed in other settings related to behavioral economics and goal setting where smooth curves over values are not observed, and instead discrete jumps in valuation before and above target values are observed.^48^ More work is needed to understand how this behavioral observation translates to risk reduction magnitudes and the reliability of scope testing in CV studies.

The overall average ratio of child-to-adult WTP was a 1.14:1 ratio, which is slightly less than high-income countries, where most values are a 1.5:1 ratio.^47^ This might be because of the differences in income between the settings. However, in both settings, people had a higher average WTP for their children than for themselves for the same risk reduction.

We had a limited convenience sample of 413 participants from health facilities in Dhaka city only. This small sample size from only health facilities is a potential limitation and source of selection bias because WTP values might have been influenced by the fact that people are already at a health facility for another illness; however, we attempted to mitigate this issue by exploring the TTT methodology so that patients could complete the survey outside the health facility. Although the derived WTP estimates are not generalizable to all of Bangladesh because of the limited sample size, we expect the findings concerning how methodological choices impact WTP to be applicable in other settings within Bangladesh and other LMICs. It was surprising that our sample had more men than women, as men tend to be the primary income earners and women the primary caregivers in Bangladesh.^28^ Data collectors explained that both spouses go to health facilities in the event of illness, and female respondents defer to their husbands when both are present at the health facility. This implies that productivity loss in households during illness is high in this context because both the primary caregiver and income earner suffer some productivity loss for a facility visit.

Data collection was delayed because of the most widespread dengue outbreak in Bangladesh since recording began in 2000.^49^^,^^50^ This outbreak could potentially affect the monetary value of WTP elicited in our results, because dengue was in the vignette example. Although this should not impact the relative comparison between methodological choices and the regression analyses since all scenarios use the dengue vignette, it could influence the overall mean VSL value. To try to remove some of the potential morbidity or disease-specific concerns, we calculated a backed-out VSL. This VSL allows us to remove the disease-specific portion of WTP and focus on the mortality risk reduction only, because all other attributes are the same across scenarios. Mean VSL using the standard method is $11 339.70 with a median of $10 413. This value is low at approximately 7 times per capita GDP of Bangladesh, which is $1698.26.^46^ When we look at the backed-out VSL, this number is even lower at $2189.52 and $3248.71 including and excluding $0 WTP values, respectively. This is much lower than elicited VSL in other settings, which typically falls between 20 and 50 times per capita GDP.^51^ More work should be done to examine why the elicited VSL value is so low in this setting, regardless of technique.

Finally, data collectors reported issues with enrolling participants. In the future, we recommend offering incentives to participants or nesting this survey in one that has direct benefits to participants.

## Conclusion

This study examines the effects that survey presentation and methodological decisions may have on results when conducting CV to elicit WTP and VSL in a LMIC such as Bangladesh. Our findings contribute to the nascent body of knowledge on approaches to improve empirical stated preference studies for WTP and VSL in LMICs. Moreover, the study also adds some of the first empirical information on the child-to-adult WTP ratio for a LMIC. In our setting individuals exhibit a higher WTP for children compared to themselves, but this difference is smaller than that observed in high-income countries. Furthermore, the results show that framing has a potentially large effect on the valuation of the intervention. Particularly, the name of the hypothetical intervention, despite equal effectiveness, has a strong influence on respondents’ WTP, with less specific language associated with lower WTP results. As such, significant care should be taken to appropriately frame the health intervention being valued within the local context to obtain a true representation of individual preferences. Consistent with the literature, we also find that offering time-to-think yielded more conservative empirical estimates, but it was challenging to implement and will likely be impractical in settings where cell phone coverage is not widespread. Additionally, further research should be conducted to examine whether individuals giving the same WTP value for different risk reductions is because of misunderstanding the probabilities or because of the small probabilities being viewed as insufficiently different from one another to warrant significant changes in investment. Finally, the VSLs calculated from our data were significantly lower when compared to local per capita GDP than in many other settings. More work should be done to determine whether this lower VSL is truly indicative of preferences or because of either income constraints or other factors related to the stated preference elicitation procedure.
